# Etiological Theories of Adolescent Idiopathic Scoliosis: Past and Present

**DOI:** 10.2174/1874325001711011466

**Published:** 2017-12-29

**Authors:** Maja Fadzan, Josette Bettany-Saltikov

**Affiliations:** 1 Scoliosis 3DC, 3 Baldwin Green Common, Suite 204, Woburn, MA 01801, USA; 2 Teesside University, Institute of Health and Social Care, Middlesbrough TS1 3BA, UK

**Keywords:** Scoliosis, Adolescent idiopathic scoliosis, Etiology, Pathogenesis, Spinal, Neuromuscular

## Abstract

Adolescent idiopathic scoliosis is one of the most common spinal deformities, yet its cause is unknown. Various theories look to biomechanical, neuromuscular, genetic, and environmental origins, yet our understanding of scoliosis etiology is still limited. Determining the cause of a disease is crucial to developing the most effective treatment. Associations made with scoliosis do not necessarily point to causality, and it is difficult to determine whether said associations are primary (playing a role in development) or secondary (develop as a result of scoliosis). Scoliosis is a complex condition with highly variable expression, even among family members, and likely has many causes. These causes could be similar among homogenous groups of AIS patients, or they could be individual. Here, we review the most prevalent theories of scoliosis etiology and recent trends in research.

## 1 WHAT IS SCOLIOSIS?

Scoliosis is a 3-dimensional deformity of the spine and trunk, which affects millions of people worldwide. While 20% of scoliosis cases can be attributed to neuromuscular, syndromic, or congenital disorders, as much as 80% of all scoliosis is termed “idiopathic” or of unknown etiology. Clinical and experimental documentation regarding the theories of etiology support the trend of many possible causes of idiopathic scoliosis. In this review, we will examine current and past theories of scoliosis etiology.

## 2 PREVALENCE OF AIS

According to current literature, the prevalence rate of AIS ranges from 0.47-5.2% [1], but it is commonly accepted as 2-3% of the general population. The prevalence and severity of scoliosis is higher in girls than in boys, with the female-to-male ratio rising from 1.4:1 in mild curves (10° to 20°) up to 7.2:1 in more severe curves (>40°) [1].

Several hypotheses have tried to account for this difference in sex distribution. One of the most plausible put forward by Schultz [2] describes a mechanical model in which it is suggested that if the progression of a lateral curve is thought of as the buckling of a spinal column then the likelihood for progression to occur is proportional to the height of the column and inversely proportional to its thickness. Put simply, tall slim spines are more likely to buckle than shorter thicker ones. Schultz confirmed this theory experimentally and demonstrated that girls’ spines were indeed more slender with narrower vertebral bodies than boys. However, whether this is the actual cause of the increased incidence of scoliosis in girls is a debatable issue. Studies have found that the prevalence of scoliosis is also higher in adolescents who participate in certain sports activities, such as dance, ballet, swimming, tennis, table tennis, hurling, javelin, volleyball, gymnastics, and rhythmic gymnastics [3-18]. Despite these findings, there is no evidence to suggest a causal relationship between scoliosis and any sports activity. Research in this area is limited as most studies are retrospective case-controls, which have a bias in retrieved information [15].

## 3 RISK FACTORS

Czaprowski *et al.* [19] found that joint hypermobility occurs more frequently in children with idiopathic scoliosis than in healthy sex and age-matched controls. Others have speculated that joint hypermobility is a risk factor for idiopathic scoliosis as it may predispose to spinal instability [20]. In a similar vein, Tanchev *et al.* [10] found a 10-fold higher prevalence of scoliosis in rhythmic gymnasts and suggested that there may be a “dangerous triad” of generalized joint laxity, delayed maturity, and asymmetric spinal loading which plays an important etiologic role in the development of scoliosis and other spinal deformities. One could hypothesize that because flexibility is an asset in certain sports like gymnastics, children who are hypermobile could be drawn to the activity, as they would excel at it, and these children may be more prone to developing scoliosis [5]. It is possible that the repetitive physical demands of sports, particularly movements that place asymmetrical loads on the spine and place the thoracic spine in a lordotic position, could accelerate an existing scoliosis or disrupt spinal mechanics in a child with a pre-existing disposition to developing scoliosis.

In addition to joint laxity, growth-related factors have also been suggested to contribute to the development of AIS. Willner [21, 22] put forward the theory in 1974 that girls with adolescent idiopathic scoliosis were taller than normal controls and that growth in the scoliosis group occurred faster in the pre-teen years than in later years. Other investigators, however, in subsequent studies did not find any abnormal growth pattern, velocity or development in patients with idiopathic scoliosis [23, 24]. 

Archer and Dickson [25] later reported that female scoliosis patients (with a ≥15° curve) were taller than girls with smaller curves. The authors suggested that these height differences could be genetic, but it could also be the flattening of the thoracic kyphosis that contributes to the discrepancy [25]. In 2014, Hershkovich *et al.* [26] found a positive association between body height and the risk for spinal deformities by severity (spinal deformities included idiopathic scoliosis and kyphosis). Though growth may or may not play a role in the etiology of AIS, it certainly affects the pathology. A 2005 study by Ylikoski [27] found that a growth velocity of more than 2 cm per year is associated with curve progression.

In females, the timing of the peak growth rate is strongly correlated to menarche [28]. Studies have shown that delayed puberty and late age at menarche are associated with higher prevalence of AIS [29-31]. Grivas *et al.* [32] reviewed the epidemiological data available on AIS prevalence rates worldwide and the average age of menarche (in normal subjects) in those locations. In that study, researchers found that menarche typically occurs later in girls that live in northern latitudes, which corresponds to higher prevalence rates of AIS. Grivas *et al.* [32] hypothesize that geography may be related to AIS pathogenesis, with latitude influencing sunlight, melatonin secretion (a hormone frequently linked to AIS), and the age at which menses occurs.

While menarcheal status is an important consideration for clinicians treating scoliosis, the weight of this one factor as a measurable risk factor for AIS and its potential role in etiology is convoluted by the fact that age at menarche can vary significantly within a population, and is influenced by a multitude of genetic, socioeconomic, environmental and lifestyle factors [33].

One such factor is body mass index (BMI). Several studies have linked relatively high BMI with earlier menarche, and low BMI with delayed menarche [34-36]. Interestingly, low BMI has also been found to be associated with AIS [15, 26, 37], as well as abnormal levels of leptin - a hormone known to play a role in fat regulation and the onset of puberty [37-39].

Perhaps a more apparent risk factor for scoliosis is family history. In 2012, Grauers *et al.* [40] estimated that the heritability of scoliosis is about 38%, using data from the Swedish Twin Registry. Watanabe *et al.* [15] found that the odds ratio for developing scoliosis was 1.5 times higher for participants whose mothers had scoliosis while Tang *et al.* [41] found that the sibling recurrence risk of scoliosis in a Chinese cohort of female AIS patients was 18%. Grauers *et al.* [42] later found that patients who had a family history of scoliosis were at a slightly higher risk of having curves requiring treatment, compared to patients who did not have a family member with scoliosis.

## 4 ANATOMICAL CHANGES OCCURRING IN SCOLIOSIS

Many etiological theories of scoliosis look to biomechanical origins, particularly concerning the relationship between the sagittal and coronal planes of the spine. However, before delving into this topic it is important to understand the anatomical changes occurring in scoliosis. Scoliosis is a complex deformity in that it not only involves a lateral curvature in the coronal plane, but occurring simultaneously is a rotational deformity of the vertebral column along the longitudinal axis as well as a lordotic deformity in the sagittal plane. The shape of the individual vertebra in structural scoliosis also undergoes significant change. On the superior and inferior border of each vertebral body is the vertebral ring epiphysis through which growth in height occurs. Asymmetric pressure on the immature vertebrae causes the vertebral section on the concave side of the curve to decrease growth [43] whereas the other convex vertebral section where less pressure is applied has normal or accelerated growth. This leads to wedging of the vertebra [44]. A translatory motion then occurs in the direction of least resistance; that is towards the convexity of the curve with the vertebral body under the most compressive force moving most laterally (apical vertebra). Associated with the translatory motion in the coronal plane is a rotatory movement of the vertebra along the transverse axis. The vertebral body rotates towards the convex side of the curve and the spinous processes rotate towards the concave side. As the vertebrae rotate and bend laterally, the discs are compressed on the concave side and distracted on the convex side of the curve. The vertebral body becomes distorted in shape towards the convex side and the pedicles, laminae and transverse processes become thicker on the convex side. Conversely, on the concave side in the thoracic region the pedicles become wafer thin and are accompanied with a narrowing of the spinal canal on this side [45].

In scoliosis, anatomical changes occur in the soft tissue structures surrounding the vertebral bodies. Shortening of these tissues occurs on the concave side of the curve. This is also accompanied by a shortening of the intervertebral joint capsule, which may lead to facet joint compression and ultimately osteoarthritis. Additionally, the intervertebral muscles, the erector spinae, the quadratus lumborum, the psoas major and minor and the oblique abdominals all shorten on the concave side. The anterior and posterior longitudinal ligaments, the ligamenta flava and the interspinous ligaments also shorten to this side, and limit flexion towards the convex side [46].

As the vertebrae rotate, the ribs, which are attached to the vertebrae by the musculoskeletal system, follow the rotational torque applied by the spine. They are pushed downwards as well as forwards on the concave side. This causes a crowding of ribs posteriorly on the concave side as well as a small hump on the anterior chest wall of the same side. Conversely, the ribs on the convex side become widely separated and are pushed backwards, creating a rib hump on the posterior chest wall. Associated with the posterior movement of the ribs is a narrowing of the rib cage on the convex side. The ribs on the convex side then push against the scapula and make it more prominent [45].

Movement of the spine laterally generally tends to cause a spinal imbalance. This means that the head of the patient does not remain centered over the pelvis, but causes the head and upper torso to fall to the left or right of the gluteal cleft causing altered spinal mechanics and subsequent degenerative joint disease [47].

## 5 BIOMECHANICAL THEORIES

Somerville [48] first described thoracic idiopathic scoliosis as a combination of lordosis, axial rotation, and lateral flexion and suggested that the lordosis arises from a failure of growth of posterior elements of a segment of the spine. Roaf [49] further described this theory in 1966, and suggested that the fundamental problem in scoliosis is the relative lengthening of the anterior components of the spine compared to the posterior structures. This situation in a stiff anterior musculoskeletal wall should result in lateral deviation of the spine and the development of scoliosis.

Lawton and Dickson [50] state that their experiments with rabbits support Roaf’s Hypothesis. The investigators developed a pure frontal plane deformity in one group of rabbits, a pure sagittal plane deformity in a second group of rabbits and a combined sagittal and frontal plane deformity in the third group. Their results demonstrated that progressive experimental scoliosis developed only in those animals that had both sagittal (lordosis) and coronal plane deformities. None of the rabbits that had a pure single plane deformity developed progressive scoliosis. They also noted that when the deformity was released before maturity in the group of rabbits who had the two-plane deformity, the deformity resolved spontaneously. The investigators support the view that the anterior structures of the spine grow faster than the posterior ones, causing a loss of normal kyphosis and a buckling of the vertebral bodies (anterior elements) outwards laterally. The authors also state that correction of the deformity in the coronal plane alone, as carried out in many surgical procedures, does not correct the loss of normal kyphosis. Ohlen’s [51] work on human subjects supports Lawton and Dickson’s work when it was demonstrated that scoliotics have less thoracic kyphosis than normal.

By using the forward bend test and observing subjects from a lateral point of view, Weiss and Lauf [52] studied the prevalence of impaired forward flexion (IFF) in children ages 2, 4, and 5 years old. They describe IFF as an area in the lower thoracic region where the arch of the thoracic spine is interrupted by a short vertebral segment that appears straight and cannot actively or passively be flexed forward Fig. (**1**). Weiss and Lauf found that IFF is less common in 2 year-olds (7.9%) than in 4 and 5-year-olds (78.9% and 70.8%, respectively) and hypothesize that the reason for this is the transition from crawling to upright posture.

Previously, Tomaschewski [53] studied IFF (in at least one motion segment) in 686 healthy 9-10-year-olds - citing a rate of 16.5%. Of that 16.5%, 27% went on to develop idiopathic scoliosis within a year of follow-up. It is possible that impaired forward flexion may resolve for most children as they adapt to walking upright. However, for those of whom IFF does not resolve, or for those who develop multiple segments of IFF, it could be a factor creating instability in the spine - leading to rotational and lateral deviation during periods of growth.

Anatomical and MRI studies in humans have now established that in patients with structural scoliosis, the anterior elements of the spine are indeed longer than the posterior elements [54-60]. This condition is commonly called ‘relative anterior spinal overgrowth’ (RASO). That said, the role of RASO and sagittal plane deformity (*i.e.* thoracic lordosis) as the primary initiating factor for AIS, rather than a secondary factor involved in progression, has been called into question and is controversial [61-67]. Moreover, this concept does not seem to apply to other curve types such as single lumbar curves.

Notably, Brink *et al.* [67] measured the difference in length between the anterior and posterior side of each vertebral body and intervertebral disc, and between the anterior side of the spine and the spinal canal for AIS patients, neuromuscular (NM) scoliosis patients and normal controls. When comparing both groups of scoliosis patients with normal controls, Brink and his colleagues [67] found that the anterior elements of the spine were longer than the posterior elements, however, this spinal overgrowth was found in both the AIS and NM patients with no measurable difference between the two groups. Therefore, they concluded that RASO is more of a generalized scoliotic mechanism rather than a causative factor in AIS. Interestingly, the anterior-to-posterior length correlated linearly with Cobb angle in both NM and AIS groups, which suggests that RASO could possibly be associated with curve progression [67].

Other experimental work has concentrated on the “Hueter-Volkmann” principle. The theory suggests that increased pressure on a vertebral epiphyseal growth plate retards its rate of growth, whereas decreased pressure across the plate accelerates growth [43]. The theory suggests that on the concave side of the curve, the epiphyseal plates have abnormally high pressures that result in decreased growth, whereas on the convex side of the curve the pressure is less, resulting in accelerated growth. Stillwell’s work [68] involved the fixation of the spine in a curved position and spinous process fixation. The fixation of the spine resulted in occasional scoliosis, whereas fixation of the spinous processes resulted in severe scoliosis with lordosis and rotation.

In keeping with the Hueter-Volkmann principle, Stokes *et al.* [69] hypothesized that asymmetric loading in a “vicious cycle” causes vertebral wedging during growth in progressive scoliosis curves. Stoke’s vicious cycle hypothesis [66] Fig. (**2**) implies that whatever the cause of scoliosis, mechanical factors become predominant during periods of rapid adolescent growth, when the risk of curve progression is greatest.

In 2006, Stokes *et al.* [66] created simulations to test the vicious cycle theory and concluded that a substantial component of scoliosis progression during growth comes from biomechanical influence. In their simulations, spinal loading asymmetry was dependent on neuromuscular activation strategy. Symmetrical spinal loading was possible, but at a higher “physiological energy cost” [66]. The authors suggest that their findings could mean that different patients with AIS may adopt different neuromuscular activation strategies, which affects their spinal loading, and can explain why some curves progress more than others [66]. Similarly, Modi *et al.* [70] proposed a tuning/balancing mechanism of the spinal column and suggested that in the growing spine, there is a period of time during which the spinal column makes an effort to balance the spine. When this effort fails, the curve will progress, or if the spine rebalances, the curve will either stabilize or regress [70]. Stokes *et al.* [66, 71] suggest the possibility that muscle rehabilitation programs could affect spinal loading by providing alternate neuromuscular activation strategies for scoliosis curves that are likely to progress. This is, in fact, the objective of scoliosis-specific exercise and rehabilitation programs such as the Schroth method [72].

Another theory, which has been written on extensively by Sevastik, is the thoracospinal concept [65, 73-76]. Sevastik [73] first did experimental studies in rabbits in 1984, suggesting that asymmetric growth of the ribs may be the primary cause of deformity in some cases of right thoracic idiopathic scoliosis. However, like RASO, the idea that asymmetric rib growth is the primary initiating factor for idiopathic scoliosis is also controversial, and the theory does not fit for all AIS curve patterns [65, 77].

Leg-length discrepancy as a possible etiological factor in idiopathic scoliosis has also been studied by several authors [78, 79]. Leg length difference was found to cause a compensatory non-progressive lumbar scoliosis, but the scoliosis was only significant in leg length discrepancies of over 3 cms. Raczkowski *et al.* [80], however, determined that even smaller leg-length discrepancies (≤2 cms) could cause a functional or non-fixed scoliosis.

Functional scoliosis is often regarded as inconsequential [81], but should not be ignored by health practitioners. Postural imbalance caused by pain, injury, muscle spasms, or other factors [82] can result in a nonstructural scoliosis that can eventually progress into a fixed scoliosis if the causative factors are not found and corrected while the patient is still growing [83]. Though growth spurts are often viewed as a risk factor for curve progression, early treatment can take advantage of growth as a corrective factor, due to remaining spinal flexibility [84]. In flexible, skeletally immature spines, it does not take much to alter spinal alignment, as even carrying heavy school bags has been shown to cause a load-induced functional scoliosis in school-age children [85].

Furthermore, it is worth noting that the “normal” spine is not perfectly symmetrical in the transverse plane. The non-scoliotic spine has been shown to demonstrate a pre-existent pattern of vertebral rotation that corresponds to the most common curve types in thoracic idiopathic scoliosis [86, 87]. Castelein *et al.* [88] have hypothesized that posteriorly directed shear forces acting on the spine may contribute to existing asymmetries in the transverse plane, and increase rotational instability by way of asymmetric loading in the transverse plane of vertebrae, intervertebral discs, and attached ligaments in accordance with the Hueter-Volkmann principle.

## 6 NEUROLOGICAL THEORIES

A large proportion of studies have centered on the possibility of a neuromuscular theory for idiopathic scoliosis. When Lerique and Lecoeur [89] demonstrated in 1951 that the two sides of a scoliotic spine demonstrated action potential differences, Riddle and Roaf [90] put forward the hypothesis that muscular imbalance was a possible cause of idiopathic scoliosis. Early electromyographic work showing evidence of increased activity on the convex side of the curve was later put forward by Weiss *et al.*, Le Febre *et al.* and Hennssge [91-93]. Alexander and Season [94], however, invalidated these results in 1978 when they demonstrated that these results were due to improper patient positioning and that it was possible to induce asymmetric motor activity in normal children by positioning their spines into an asymmetric posture [95]. Other authors’ findings demonstrating fibrillation potentials in 50% of scoliotic spines [96, 97] were also invalidated by Alexander and Season who clearly showed their results were caused by noise in the system, and suggested that action potential differences were not the cause but the result of asymmetric positioning of the spine. Butterworth and James [98] supported Alexander and Season’s study [94] when they reported that the spine becomes silent when surgically fused or braced.

Early histological work put forward by James *et al.* [99] did not reveal any objective results in support of the neuromuscular theory. Hirano [100], however, demonstrated that there were clear signs of dystrophy and atrophy in the back muscles and disproportions of slow twitch versus fast twitch fibres with greater numbers of the former on the apex of the convex side [101]. Other investigators confirmed the presence of muscular abnormalities but could reveal no particular side or location [102, 103]. Differences in proportions of Type 1 and Type 2 fibres have also been located in contralateral deep muscles [102, 104, 105].

The large majority of animal work has focused on factors affecting either the growth or the stability of the spine. The excision or release of deep and superficial muscles resulted in a paralytic type of scoliosis convex to the operative site [106]. Other experimenters investigated the effect of muscle denervation. Both Liska [107] and Macewan [108] in two independent studies demonstrated that the division of the anterior and posterior nerve roots created a spinal curvature. They suggested that the interruption of the normal reflex arc was an important factor. Alexander and Season [94], however, queried the studies of Liska and Macewan when they replicated their studies. They reported that whereas all animals who had anterior and posterior nerve roots excised developed a scoliosis, only 60% of animals who had only the posterior nerve root cut developed a curve. They concluded that the final common pathway remained the efferent supply to the muscles and produced a paralytic type of scoliosis [94].

A few experimenters have also resorted to the investigation of an equilibrial cause for a neuromuscular dysfunction. The hypothesis being that idiopathic scoliosis results secondary to a disturbance at the brainstem level where impulses from the labyrinth, proprioceptive and visual systems are integrated. Yamada and co-workers [109] in 1969 noted that equilibrial abnormalities were more prevalent in idiopathic scoliosis. The equilibrial dysfunction disappeared when the subjects matured and was directly related to the severity of the curve.

Sahlstrand and associates [110] (1979) reported an increased occurrence of spontaneous and positional nystagmus in patients with adolescent idiopathic scoliosis but observed no correlation with curve size or erect and supine postures. The authors suggested that a possible feedback was occurring as a result of a deformed spine [110]. Results from histological studies are specific to severe scoliosis and must be interpreted with caution as most specimens are taken during surgical correction of severe curves, many of which had undergone traction before treatment. It is well established that treatment that disrupts muscles or ligaments by tension can disrupt central and peripheral nervous systems [108]. In 2005, Mirovsky *et al.* [111] prospectively studied thirty-one patients with severe AIS and found that only a few had vestibular and postural dysfunction, suggesting that the dysfunction occurred as a result of the patient’s misbalance. A 2015 review concluded that while there is significant evidence to suggest an association between vestibular dysfunction and AIS, animal studies have been more promising than human studies and additional research is needed in this area [112].

The thrust of most experimental work has been the recreation of a degree of imbalance in the neurological, osseous or ligamentous structures of the spine, the hypothesis being that any imbalance resulting in a scoliotic pattern may be indicative of a possible etiological factor. White [113] suggests that the presumption in these experimental works is that scoliosis is caused either by a weakness or absence of a structure on the convex side of the curve or an overactivity of the antagonist structure on the concave side.

Other researchers have postulated that in idiopathic scoliosis, there is disproportional growth occurring between the skeletal and neural systems, due to the spinal cord being short or because of a rapid growth spurt of the spine. This concept was first put forward by Roth [114, 115], and then Porter [116, 117], and has been called by several names, including uncoupled neuro-osseous growth, and now referred to as asynchronous neuro-osseous growth [118-122]. Chu *et al.* [123, 124] examined the Roth-Porter concept with MRI imaging and found that in severe AIS, the vertebral column is significantly longer compared to normal controls, but there is no detectable change in spinal cord length. Chu *et al.* suggested that anterior spinal overgrowth stretches the spinal cord and cauda equina, leading to hypokyphosis and deformity of the growing thoracic spine - causing scoliosis [123, 124].

This stretching of the spinal cord is commonly referred to as “ tethering” or “tethered cord syndrome” (TCS). It is possible that there are scoliosis patients who are considered to be idiopathic cases, but could have spinal cord tethering as an underlying pathology. The majority of idiopathic scoliosis patients do not undergo MRI, unless they have early-onset scoliosis, present with a severe curve of sudden onset, present with neurological findings, an atypical curve pattern (*i.e.* left thoracic curve), have pain, or are being screened prior to surgery [125]. Furthermore, according to Barutçuoğlu *et al.* [126], the absence of MRI findings does not definitively exclude TCS. The authors point out that somatosensorial evoked potentials or SSEP is an important additional guidance in making a diagnosis of tethered cord syndrome [126].

The identification of certain neurological conditions associated with scoliosis, such as syringomyelia, tethered cord syndrome, and Chiari malformation [127, 128] is important with respect to developing the scoliosis treatment plan. Most surgeons advocate decompression of the Chiari I malformation and syringomyelia to promote curve resolution and reduce the risk of neurologic complication, while others disagree [129]. Neurosurgical release of the filum terminale has been found to reduce curvature in scoliosis patients with TCS, syringomyelia, and Chiari malformation [130], though retethering post-surgery has also been documented [131]. Systematic extracorporeal therapy and external spinal manipulation have also been suggested as potential treatments for patients who have functional tethering of the spinal cord, however, additional research is needed in this area [132, 133].

## 7 EVOLUTIONARY THEORY OF SCOLIOSIS

It has been suggested that scoliosis has an evolutionary basis and may be the result of a selection for bipedalism in humans [134, 135]. A survey of a large sample of ape skeletons by Latimer did not find any cases of scoliosis in chimpanzees or gorillas and Lowe *et al.* [136] concluded, “naturally occurring scoliosis in vertebrates is seen almost exclusively in humans.” Lovejoy [137] proposes that this may be attributed to the difference in anatomy between humans and other apes. More specifically, humans have a longer, more mobile lumbar spine that may be more susceptible to deviation. Lovejoy [137] acknowledges that while this does not account for thoracic curvatures, a subtle imbalance in the lumbar spine could play an initiating role until more cranial effects became prominent due to other biomechanical forces.

## 8 ANIMAL STUDIES

Gorman and Breden [138] challenge this notion, suggesting that this theory of bipedalism has been reinforced because of the animal models used to study scoliosis. Pinealectomy (which creates melatonin deficiency) has been shown to cause idiopathic-type scoliosis curvatures in chickens [139], but not quadrupedal animals [140], except in the case of rats and mice that have been forced to be bipedal by amputation of their front legs and tails [135, 141].

Interestingly, in a study by Machida et al [135], researchers found that bipedal rats developed cervicothoracic lordosis, whether or not they underwent pinealectomy. The bipedal rats that did undergo pinealectomy, however, developed a lordoscoliosis similar to human idiopathic scoliosis. The researchers suggest that there may be a “disturbance of equilibrium and other postural mechanisms secondary to a deficiency of melatonin after pinealectomy which may promote the development of lordoscoliosis with vertebral rotation, especially in the bipedal posture” [135].

Despite their findings, the role of melatonin in the pathogenesis of scoliosis remains unclear. Moreover, animal models have many limitations when it comes to understanding the etiology of idiopathic scoliosis in humans, especially in light of the fact that considerable intervention is required to induce their spinal deformities. Such methods include tethering, intercostal nerve resection, electrostimulation, irradiation, pinealectomy, magnet implantation, direct injury to the epiphyseal plate, oxygen deficiency, dietary deficiency, unilateral labyrinth stimulation, plaster cast immobilization, and various local procedures which damage the spinal, neural, and/or surrounding tissues [142-145].

Janssen *et al.* [146] point out that the human spine is less rotationally stable than any other animal used in scoliosis research and that much less is required “in terms of a disturbance of the locomotor, proprioceptive, neuromuscular, or collagen metabolism systems to initiate a decompensation into a rotatory deformity in man.” They conclude that the lack of an animal model that biomechanically resembles the human spinal load is a major obstacle in scoliosis etiology research.

While this is certainly true, recent research points to fish as being a potentially beneficial model for studying idiopathic scoliosis. Gorman *et al.* [147] studied the curveback guppy as the first model for human IS demonstrating spinal curvature in healthy fish without being induced or caused by congenital vertebral malformation. Though fish do not have a bipedal gait, Gorman suggests that the biomechanical forces acting on the human and guppy spine could be similar. In both humans and guppies, the biomechanical force on the spine is along the cranio-caudal axis, with gravity acting vertically on the former, and the power of the tail-beat motion (which pushes the guppy through dense water) acting on the latter [147]. Interestingly, Gorman *et al.* found many similarities between the curveback syndrome and human AIS, including a bias for severe curvature in females (despite equal rate among the sexes), stabilization at sexual maturity, incidence of self-resolving curves, changes in vertebral shape at the apex of severe curves and variation in: curve magnitude, morphology, age of curve onset, and the rate of progression [147].

In a later study, Gorman and colleagues [148] identified a qualitative trait locus (QTL) controlling curve susceptibility in the guppy model. The locus contains over 100 genes, including MTNR1B (melatonin receptor), which is a candidate gene for human idiopathic scoliosis [149].

In fact, humans and fish share many developmental pathways and genetic similarities, which could be advantageous in furthering scoliosis etiology research. Many gene sequences isolated in fish have corresponding sequences in humans, including those involved in osteoblast and chondrocyte differentiation, bone and muscle formation, and pineal gland development [150].

In addition to guppies, zebrafish have also been studied extensively as a model for idiopathic scoliosis. AIS-like scoliosis is shown in zebrafish with mutations of protein tyrosine kinase 7 (ptk7) [151] and kinesin family member 6 [152]. Overexpression of the LBX1 (ladybird homeobox 1) gene, which has been associated with AIS in human studies, is shown to cause body axis deformation in zebrafish [153]. Analysis of ptk7 mutant zebrafish point to cilia motility and cerebrospinal fluid flow defects as the underlying biological cause of spinal curvature [154], which could potentially have implications for future human research.

## 9 GENETIC FACTORS: HUMAN RESEARCH

A genetic etiologic basis for idiopathic scoliosis has been favorably viewed since the 1920s when the deformity was first described in twins and families [155, 156]. Later studies confirmed the familial nature of this condition [157-159]. Whilst an increased incidence of the deformity was found in relatives of patients, controversy remains as to whether the condition is of dominant or multiple gene inheritance [160] or dominant and sex-linked with variable expressivity and incomplete penetrance [158]. An interesting study by Kruse *et al.* [161] supports the presence of the Carter effect in AIS. The Carter effect involves a polygenic threshold model with sex dimorphism of inheritance, with a greater genetic load (*i.e.* susceptibility genes) required for males to be affected with AIS, which, in turn, makes them more likely to transmit AIS to their children [161].

Part of the difficulty in determining the genetic background of AIS lies in phenotyping and study design. While linkage studies may seem like a logical choice due to the familial nature of scoliosis, these studies are better for finding variants in rare diseases and subphenotypes, and thus may not necessarily be effective for detecting common variants in the general population [162]. Genome-wide studies are expensive and yield large amounts of data while candidate-gene studies, though simpler, depend on the initial hypothesis and are not suitable for searching for new genes [162]. In recent years, case-control association studies are being more widely used [163].

Thus far, genetic research on AIS has pointed to many potentially associated genes, including: MATN1 (matrillin 1), TIMP2 (tissue inhibitor of metalloproteinases 2), MMP3 (matrix metalloproteinase-3), ESR1 (estrogen receptor alpha), ESR2 (estrogen receptor beta), IL6 (interleukin 6), CALM1 (calmodulin 1), VDR (vitamin D receptor), MTRN1B (melatonin receptor type 1b), CDH7 (cadherin 7), TPH1 (tryptophan hydroxylase 1), TNFRS11B (tumor necrosis factor receptor superfamily member 11b), GPER (G protein-coupled estrogen receptor 1), IGF1 (insulin-like growth factor 1), HSPG2 (heparan sulfate proteoglycan 2), FBN1 (fibrillin-1), FBN2 (fibrillin-2), COL11A2 (collagen type XI alpha 2 chain), LBX1 (ladybird homeobox 1), GPR126 (G-protein coupled receptor 126), BCN2 (basonuclin-2), PAX1 (paired box 1), TGFB1 (transforming growth factor beta 1), DOT1L (disruptor of telomeric silencing 1-like), IL-17RC (interleukin 17 receptor C), C17orf67 (chromosome 17 open reading frame 67), POC5 (POC5 centriolar protein), NUCKS1 (nuclear casein kinase and cyclin dependent kinase substrate 1), ZIC2 (zinc finger protein ZIC 2), FAM101A (regulator of filamin protein A), COMP (cartilage oligomeric matrix protein), PITX1 (paired like homeodomain 1), and homeobox genes HOXB7, HOXB8, HOXA13, and HOXA10 [149, 164-198].

Of these, gene variants rs11190870 downstream of the LBX1 gene, rs657507 on GPR126 intron, and rs12946942 on chromosome 17q24.3 near the genes SOX9 and KCNJ2, have been replicated in additional studies [162]. Though there has been a lot of development in AIS genetic research in recent years, genetic heterogeneity continues to be an obstacle. Future studies with larger cohorts are needed to make any sort of clinical impact in identifying who is susceptible to scoliosis and which AIS patients are at greater risk for progression. Studying familial AIS in younger unaffected siblings of AIS girls in a longitudinal study may be another way to advance our knowledge [199]. Prognostic DNA testing for scoliosis and blood testing for scoliosis susceptibility have already been developed [200-202], but the validity of these methods requires further evaluation [203-205].

Twin studies have established a higher concordance rate in monozygotic twins versus dizygotic twins [206-210]. That said, phenotypic variability (*i.e.* different expression of curve pattern, severity, *etc.*) exists among affected family members, and even among monozygotic twins, suggesting that environmental factors are also at play [40, 211-214]. It is possible that phenotypic differences in monozygotic twins could be the result of epigenetic differences that accumulate over time [215, 216]. Epigenetics is defined as heritable changes in gene expression without a change in underlying DNA sequence [217]. Epigenetic changes can occur normally as a part of development, but can also be influenced by external environmental factors including diet, exercise, certain chemicals and medications [218, 219]. DNA methylation, histone modification and nucleosome positioning, and noncoding small RNAs are molecular mechanisms that have been found to have an effect on gene expression [217]. Burwell *et al.* [220] have suggested that new research is required to look for chromatin modifications in AIS subjects and vertebral growth plates excised at surgery.

## 10 ENVIRONMENTAL FACTORS

Goldberg *et al.* [221, 222] suggested that scoliosis is caused by environmental stress causing developmental instability. Environmental factors could be hormonal, nutritional, alcohol, smoking, viruses, drugs, medications, toxins, and physical activity [220]. Additionally, Hawes and O’Brien [223] have noted that scoliosis has occurred in children in response to psychological distress, trauma, back injury, surgery, cancer treatment (radiation and chemotherapy), infections, tumors, and birth injuries.

 In 1980, Pratt and Phippen [224] found increased levels of copper in hair samples of AIS patients and suggested that copper may be a factor in the development of scoliosis since it is part of the lysyl oxidase enzymes required for cross-linking of collagen and elastin. Dastych *et al.* [225] also found increased levels of copper in hair samples of AIS patients, along with increased levels of zinc and decreased levels of selenium. In a separate study, zinc concentration in hair and serum in AIS subjects was similar to controls, but the back muscles of scoliosis subjects undergoing surgery were found to contain decreased zinc [226]. The authors concluded that this is likely a secondary change, rather than one of primary etiological importance [226].

Webb *et al.* [227] and Green *et al.* [228] discovered virus-like particles in the paraspinal muscles of scoliosis patients in 1976 and 1979. However, the significance of these findings is unclear and, to the best of the authors’ knowledge, has not been investigated or confirmed in more recent studies.

Worthington and Shambaugh [229] suggested that nutritional deficiencies might play a role in the etiology of AIS. Chlebna-Sokol *et al.* [230] found that in a study of 74 children with skeletal abnormalities (including scoliosis, bone fractures, Scheuermann’s disease and thorax deformations), all subjects had significantly low vitamin D intake and the majority also had calcium deficiency. In the same group, 14/74 subjects had either osteopenia or osteoporosis; however, the authors did not find any significant correlations between the skeletal diseases and abnormalities in the diet [230]. Balioglu *et al.* [231] and Batista *et al.* [232] also found that AIS patients are deficient in Vitamin D, and other researchers have linked inadequate calcium intake with osteopenia in AIS patients in an Asian population [233, 234]. While a low calcium intake is known to aggravate vitamin D deficiency [235], additional research is required to determine if dietary changes can have any effect on AIS, as environmental factors acting on scoliosis are currently poorly understood.

## 11 HORMONAL FACTORS

In an attempt to develop a multifactorial theory of AIS etiology, Burwell *et al.* [199] put forward the “Cascade Concept” based on the earlier findings of Clark *et al.* [236] and other researchers who found an association between AIS and low leptin levels [37-39]. Clark *et al.* [236] carried out a population-based prospective study which determined that low fat mass, low lean mass, low circulating leptin and high circulating adiponectin levels in 10-year olds are associated with scoliosis found at 15 years old. Burwell *et al.* [199] speculate that leptin plays a role in central nervous system (CNS) development and that lower levels of leptin are responsible for initiating asynchronous neuro-osseous growth, causing tension in the neuraxis. The authors suggest that neuraxis tethering is not expressed caudally at the conus level, which has been found to be normal in AIS patients [237, 238], but cranially in the upper cervical cord and medulla oblongata (as disturbed white matter) and at the craniocervical junction (as low-lying cerebellar tonsils), in accordance with findings by Kong *et al.* [239] and Chu *et al.* [240]. Once a spinal deformity has been initiated, other biomechanical or hormonal disturbances, especially those that cause reduced vertebral bone mass, may lead to curve progression [199].

As mentioned earlier, melatonin has been a hormone of interest in the study of AIS ever since it was discovered that melatonin-deficient animals could develop scoliosis [241], however, human studies have shown mixed results. Machida *et al.* [242] found significantly decreased melatonin levels in adolescents with progressive scoliosis as compared to patients with stable curves and normal controls. Sadat-Ali *et al.* [243] also found lower melatonin levels in AIS patients versus controls. Hilibrand *et al.* [244], Fagan *et al.* [245] and Bagnall *et al.* [246] did not find a significant difference in nighttime or daytime melatonin levels between AIS patients and normal controls. These conflicting results led to the proposal that AIS is instead caused by a melatonin-signaling pathway dysfunction that only affects certain cell types, namely osteoblasts [247, 248].

Melatonin plays a complex role in human biology [249]. As it relates to scoliosis, melatonin is believed to be involved in the onset of puberty [250], and thought to have a protective effect on bones, by preventing degradation and promoting bone formation [251-253]. Additionally, melatonin serves as an antagonist for calmodulin - a calcium-binding receptor protein that regulates smooth muscle contraction [255]. Studies on AIS patients have shown increased levels of calmodulin in platelets (when compared to normal controls) [256] and asymmetrical distribution of calmodulin in paraspinal muscles, with increased levels at the convexity of the curve [257]. In a study by Lowe *et al.* [256], platelet calmodulin levels correlated closely with curve progression. However, it has been suggested that the increasing calmodulin levels do not play an etiologic role in AIS and simply reflect changes in cellular calcium and sarcomere metabolism related to changes in muscle contractility associated with curve progression [258]. In light of the fact that osteopenia has been reported to be either a causative or co-existing factor in AIS [31, 233, 234, 259-267], the relationship between scoliosis, melatonin signaling, and bone integrity warrants further investigation.

Additionally, since scoliosis curve progression is linked to puberty, and females are more likely to progress than their male counterparts, much research has focused on growth and sex hormones. Several researchers have found that growth hormone levels in children with idiopathic scoliosis are higher than in controls [268-270], whereas Misol *et al.* [271] did not find a difference.

Kulis *et al.* [272] found that levels of FSH (follicle-stimulating hormone), LH (luteinizing hormone) and oestradiol were lower in premenarcheal AIS patients than in normal premenarcheal girls, while higher levels of progesterone, oestrone, oestriol, RANKL (receptor activator of nuclear factor kappa-B ligand), osteocalcin and AP (alkaline phosphatase) were observed in the group of AIS patients. Skogland *et al.* [268] and Raczkowski [273] found elevated levels of testosterone in AIS patients, both in prepubertal girls and in older teenage girls. Contrarily, Esposito *et al.* [274] found lower levels of testosterone, progesterone, and 17-beta-estradiol (oestradiol) in AIS patients.

Testing serum levels in AIS patients may be an inadequate measure as Pollanen *et al.* [275] recently proved that 17-beta-estradiol is synthesized by muscular cells and suggested that systemic levels of sex steroid hormones may not follow the same trend as levels found in skeletal muscle. Rusin *et al.* [276] found asymmetric expression of estrogen receptor 2 (ESR2) in deep paravertebral muscles more on the side of the convexity than the concavity. As with most other findings associated with AIS, whether or not these differences in expression are primary or secondary to the condition remains to be solved.

## CONCLUSION

White [113] sums up all the hypotheses relating to the etiology of scoliosis as follows:

“The normal spine in a growing person has a precise, precarious, delicate mechanical balance. Asymmetrical changes in primary structures, support structures, growth centres, the position of the spine and related neural or muscular components can result in the development of scoliosis.”

Currently, idiopathic scoliosis treatment is not rooted in causality but instead aims to prevent further progression by biomechanical intervention (*i.e.* bracing, surgery). The principle behind exercise rehabilitation programs is postural re-education to reduce asymmetric spinal loading during growth, though these programs are not yet widespread standard treatment. Adjunct treatments for patients presenting with osteopenia and/or hypermobility may be helpful in curbing progression, however, there is no standard testing done to identify these associated conditions in AIS patients. MRI studies, while costly, could potentially identify subclinical neurological abnormalities in AIS patients and further our understanding of etiology.

It is generally accepted that earlier intervention in AIS is preferable, but when mild AIS is first diagnosed, there is little effort made to determine the underlying cause and treatment is usually not recommended until the scoliosis proves to be progressive [223]. There is great interest in DNA-based tests to determine which patients are at risk of developing scoliosis and which patients with scoliosis are most likely to progress, however, the current understanding of the relationship between genetic factors and environmental factors in the development and pathogenesis of AIS remains limited. Since scoliosis is a complex, multifactorial condition, the continued effort and collaboration of professionals across multiple disciplines is needed to further knowledge in this field. The tendency to group all AIS patients into one theory is perhaps a hindrance in moving forward.

## Figures and Tables

**Fig. (1):**
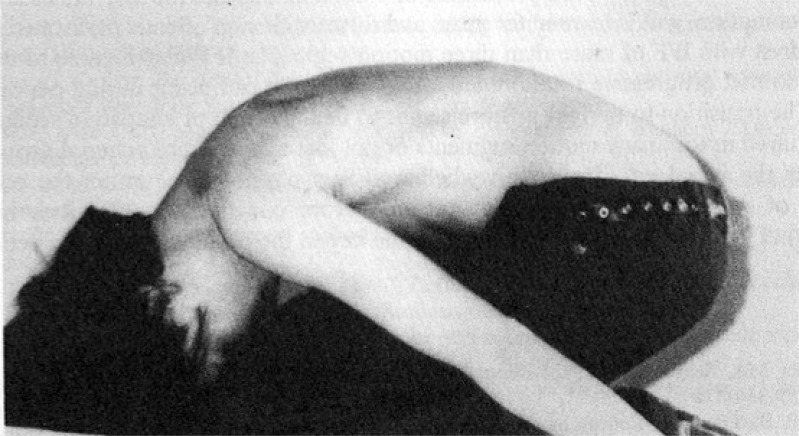
Impaired Forward Flexion (adapted from [52]).

**Fig. (2):**
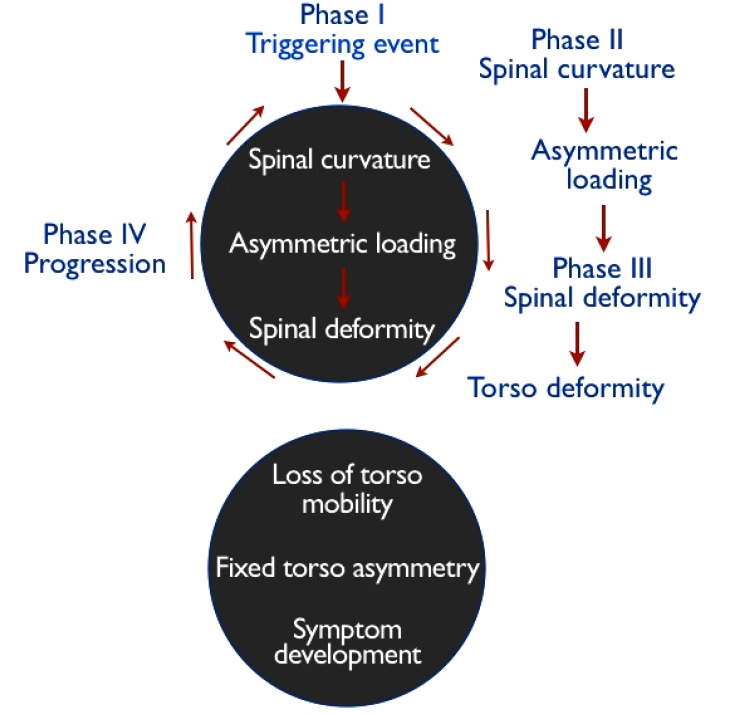
Stoke’s Vicious Cycle of Pathogenesis: A lateral spinal curvature produces asymmetrical loading of the skeletally immature spine, which in turn, causes asymmetrical growth and a progressive wedging deformity. Adapted from, “Scoliosis and the Human Spine” by Martha C. Hawes (2002).
